# The role of invariant surface glycoprotein 75 in xenobiotic acquisition by African trypanosomes

**DOI:** 10.15698/mic2023.02.790

**Published:** 2023-01-27

**Authors:** Alexandr Makarov, Jakub Began, Ileana Corvo Mautone, Erika Pinto, Liam Ferguson, Martin Zoltner, Sebastian Zoll, Mark C. Field

**Affiliations:** 1 School of Life Sciences, University of Dundee, Dundee, DD1 5EH, UK.; 2 Laboratory of Structural Parasitology, Institute of Organic Chemistry and Biochemistry, Czech Academy of Sciences, 16610 Prague 6, Czech Republic.; 3 Laboratorio de Moléculas Bioactivas, Departamento de Ciencias Biológicas, Universidad de la República, Paysandú 60000, Uruguay.; 4 Charles University, Faculty of Science, Department of Parasitology, Vestec, Czech Republic.; 5 Institute of Parasitology, Biology Centre, Czech Academy of Sciences, 37005 Ceske Budejovice, Czech Republic.

**Keywords:** invariant surface glycoprotein, trypanosome, suramin, drug metabolism, drug accumulation, CRISPR/Cas9, xenobiotics

## Abstract

The surface proteins of parasitic protozoa mediate functions essential to survival within a host, including nutrient accumulation, environmental sensing and immune evasion. Several receptors involved in nutrient uptake and defence from the innate immune response have been described in African trypanosomes and, together with antigenic variation, contribute towards persistence within vertebrate hosts. Significantly, a superfamily of invariant surface glycoproteins (ISGs) populates the trypanosome surface, one of which, ISG75, is implicated in uptake of the century-old drug suramin. By CRISPR/Cas9 knockout and biophysical analysis, we show here that ISG75 directly binds suramin and mediates uptake of additional naphthol-related compounds, making ISG75 a conduit for entry of at least one structural class of trypanocidal compounds. However, ISG75 null cells present only modest attenuation of suramin sensitivity, have unaltered viability i*n vivo* and *in vitro* and no alteration to suramin-invoked proteome responses. While ISG75 is demonstrated as a valid suramin cell entry pathway, we suggest the presence of additional mechanisms for suramin accumulation, further demonstrating the complexity of trypanosomatid drug interactions and potential for evolution of resistance.

## INTRODUCTION

The surfaces of infectious agents support multiple functions, including cellular invasion, defence from innate and acquired immune responses, environmental sensing and nutrient uptake. African trypanosomes possess a completely extracellular life cycle, therefore immune evasion is critically important and is mediated by a highly sophisticated mechanism of antigenic variation [1]. This process leads to periodic replacement of the variant surface glycoprotein (VSG) that dominates the surface, with an immunologically distinct paralog. Embedded within this coat are transporters, signalling proteins and receptors, together with a poorly understood, but highly expressed, superfamily of invariant surface glycoproteins (ISGs), which share the VSG structural architecture [2–5]. A considerable repertoire of proteins grouped into specific subdomains is present on the surface of *Trypanosoma brucei*, indicating a high level of organisation, including life stage-specific expression and localisation [6]. ISGs are expressed from multi-gene arrays by most mammalian stage African trypanosome subspecies. However, most paralogs are almost identical at the sequence level, and ISGs do not exhibit antigenic variation. The abundance of some ISG subfamilies is mediated by ubiquitylation, suggesting potential environment/ligand-dependent regulation [7, 8]. ISGs have considerable presence within the endosomal system in addition to the surface and are actively recycled, with their abundance consistent with potential accumulation of material from the host environment [9].

ISGs are type I trans-membrane domain glycoproteins, initially identified via surface iodination and expression library screening [10–13]. Following sequencing of the genome we identified five distinct ISG subfamilies: ISG65, ISG75 and additional subfamilies ISGL, ISGLA and ISGLB [2]. Significantly all five subfamilies are conserved across the African trypanosome group but absent beyond it; structural similarities between ISGs and VSGs suggest a common origin, and probably that ISGs arose prior to antigenic variation [14]. ISGs are considerably less abundant than VSG, at ∼10^4^ copies per cell against ∼10^7^ for VSG, but are the second most abundant known proteins on the trypanosome surface. Despite being invariant, ISGs are not candidates to vaccinate against African trypanosomes, although a clear and robust immune response has been successfully exploited for diagnosis [15], likely due to combined antibody shielding by VSG and rapid endocytosis removing immune effectors from the surface [14]. Notably, ISGs are exclusively expressed in the mammalian infective form, anticipating a specific role within this life stage [10, 11].

The full range of physiological ligands recognised by ISGs remains undetermined: ISG65 was recently characterised to bind complement component C3b [16], while ISG75 was previously identified as a suramin-sensitivity protein through a genome wide screen [17]. Suramin, a polysulfonated trypan blue derivative, is one of a series of multi-naphthalene ring compounds synthesised over a century ago [18, 19] with a variety of potential clinical applications, and remains in use for treatment of *T. brucei rhodesiense* infection [20]. A high negative charge precludes passive diffusion across biological membranes, hindering access to the central nervous system (and hence utility for late-stage disease), suggesting a need for a specific uptake mechanism. Suramin is also valuable for treatment of surra caused by *Trypanosoma evansi* [21], is active against *Trypanosoma cruzi* [22, 23], *Leishmania major, Leishmania donovani* and *Plasmodium falciparum* [24, 25] and used to treat river blindness caused by *Onchocerca volvulus* [26]. In addition to anti-parasitic applications, suramin has been trialled against a range of viruses including hepatitis, herpes simplex, human immunodeficiency virus, ebola, zika and chikungunya [27–31]. Suramin also has impact on autism spectrum disorder [32], and has been suggested as a chemosensitizer for cancer treatment [33–35].

Mechanisms behind suramin uptake and toxicity in trypanosomes remain unclear. Several suramin sensitivity genes were identified in a genome wide screen, including the lysosomal major facilitator superfamily transporter (MFST), ISG75, components of the AP-1 adaptin complex and several lysosomal proteins [17]. Of these, ISG75 was proposed as the surface receptor for suramin, and knockdown indeed decreases suramin binding and uptake [17, 36]. Strengthening this hypothesis, we also demonstrated that suramin uptake proportionally rises with ISG75 expression level [37]. However, ISG75 knockdown did not follow this trend, with an 80% ISG75 depletion conferring just a 25% drop in uptake [37], resulting in only mild two to three-fold decreased suramin sensitivity [17, 37]. By comparison MFST knockdown confers a ten-fold increase in suramin resistance, while expression of a recently identified variant surface glycoprotein, VSG^sur^ confers a more impressive 100-fold increase in resistance [17, 38].

The differential impact of ISG75 silencing versus VSG^sur^ expression asks if the modest effects of the former are related to residual expression, and that complete depletion would lead to a more prominent effect; or whether alternative uptake pathways exist for suramin in trypanosomes. One such pathway may be via interactions with low density lipoprotein (LDL): suramin has promiscuous interactions with serum proteins [37], and specific serum components appear to influence suramin accumulation. Specifically, suramin uptake correlates with LDL levels in media, suggesting that the two pathways are linked in some manner [39]. Notably, the LDL receptor is uncharacterised in trypanosomes and involvement of ISG75 in LDL uptake uninvestigated, albeit that alterations to endocytic pathway dynamics differentially impact LDL accumulation and suramin sensitivity, arguing against a link [40, 41]. Lastly, we recently demonstrated that suramin elicits up-regulation of quiescent form differentiation markers and activation of Krebs' cycle enzymes [37]. Notably, VSG^sur^ has nanomolar affinity for suramin [42] and is proposed to act as a sink, sequestering suramin both at the cell surface and within the endocytic pathway. In light of these observations, we questioned if suramin uptake via ISG75 plays a principal role in eliciting these changes.

We used CRISPR/Cas9 to generate a null cell line and performed direct *in vitro* biophysical measurements of suramin interactions with recombinant ISG75. We find evidence that suramin binds ISG75 directly, that ISG75 is involved in accumulation of addition structurally related trypanocides but, surprisingly, has little contribution to viability in *in vitro* culture or infectivity in a mouse model. Further, we confirm modest alterations to the EC_50_ of suramin as initially observed by RNAi [17], suggesting an additional pathway(s) for suramin activity against trypanosomes is most likely present.

## RESULTS

### ISG75 directly binds suramin and trypan blue

**Figure 1:**
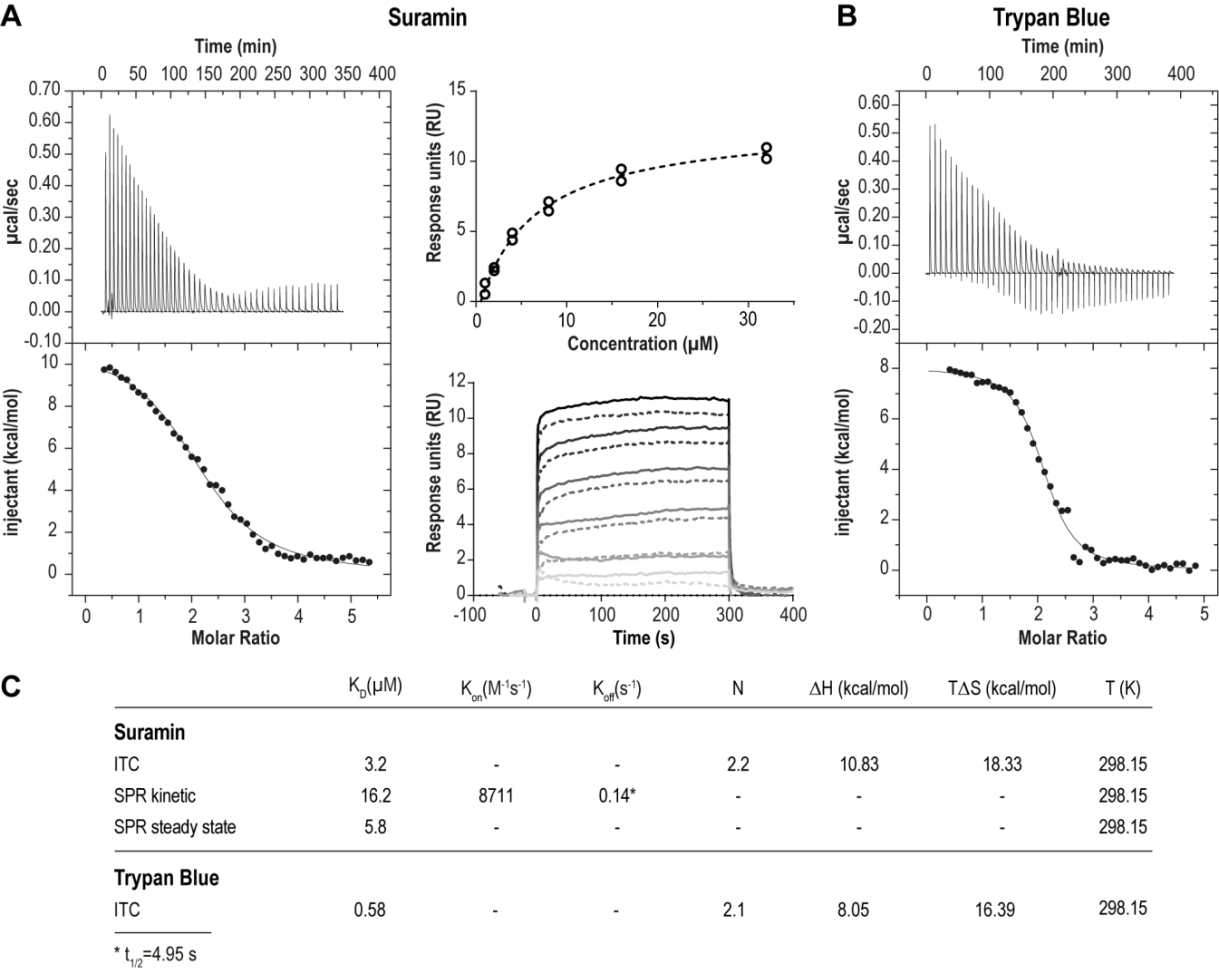
FIGURE 1: ISG75 binds suramin and trypan blue directly. **(A)** Left panel – Isothermal titration calorimetry of the interaction of suramin with ISG75. 400 µM suramin was titrated into 16 µM ISG75 solution within the calorimetric cell. The upper left panel shows the raw data of sequential suramin injections, the lower panel the integral after subtraction of the heat of suramin dilution (dots) together with the fit (line). Right panel – Surface plasmon resonance of the ISG75:suramin interaction. Avi-tagged ISG75, site-specifically biotinylated at the C-terminus was used as ligand and immobilised onto the CAPture sensor chip surface. Suramin was used as analyte and flowed over the chip at 1, 2, 4, 8, 16 and 32 µM. Data were reference-subtracted and fitted using steady state (upper right) and one-to-one kinetic analysis (lower right). For fitting, data points from two independent measurements (circles, upper panel; solid/dashed lines, lower panel) were used. **(B)** Isothermal titration calorimetry of the interaction of trypan blue with ISG75. 500 µM trypan blue was titrated into 16 µM ISG75 solution within the calorimetric cell. The upper left panel shows the raw data of sequential trypan blue injections, the lower panel the integrated heats after subtraction of the heat of trypan blue dilution (dots) together with the fit (line). **(C)** Kinetic and thermodynamic parameters of the ISG75:suramin and ISG75:trypan blue interaction. K_D_, dissociation constant; K_on_, association rate; K_off_, dissociation rate; N, stoichiometry; ΔH, change in enthalpy; ΔS, change in entropy; T, temperature in Kelvin; t_1/2_=complex half-life time.

We first assessed the physical interaction between suramin and ISG75. Using isothermal calorimetry (ITC) and surface plasmon resonance (SPR) we found evidence for direct binding between recombinant ISG75 and suramin (**Fig. 1**). The extracellular domain (residues 29 - 462) of *Trypanosoma brucei gambiense* ISG75 was expressed and obtained tag-free from insect S2 cells or with a biotinylated C-terminal Avidin-tag from human Expi293F cells and used as bait for ITC and SPR respectively (Fig. S1A). ITC-measured dissociation constants (K_D_) for suramin are 3.2 µM (**Fig. 1A** left, Fig. S1B). For context, free suramin concentrations in patient blood during treatment are estimated at ∼15 µM [39, 43] and thus sufficiently high to facilitate uptake via ISG75. For the related compound trypan blue, K_D_ was measured at 0.6 µM (**Fig. 1B**). ITC also suggests binding of two molecules of suramin or trypan blue per ISG75 molecule. For binding both suramin and trypan blue the entropic contribution (ΔS) is greater than the enthalpic (ΔH), suggesting an entropy driven interaction and predominantly hydrophobic and temperature dependent. At physiological temperature the Gibbs free energy ΔG is negative, indicating an endothermic and hence thermodynamically favourable interaction (**Fig. 1C**). While we assume a simultaneous binding model (two molecules of suramin could bind at once and independently of each other), sequential binding with different affinities for each site remains possible but has not been investigated.

Using small angle X-ray scattering we found that the extracellular domain of ISG75, unlike homodimeric VSGs [44] and heterodimeric *T. brucei* transferrin receptor [45], presents as monomeric and that ligand-binding does not change the oligomeric state (Fig. S1C, D). However, this applies only to the isolated extracellular domain, and other data from us suggest that ISG75 is dimeric, implicating interactions mediated by the coiled coil domain in the extracellular C-terminal domain [2]. Note that for drug binding assays we used the non-coiled coil fragment of the ISG75 extracellular domain that is uninvolved in dimerisation, and we assume that dimerisation is unlikely to substantially impact suramin interactions.

To model the orientation of ISG75 on the cell membrane we also measured suramin binding to surface-immobilised ISG75 using SPR, where the ISG75 fragment was attached to the substrate via the biotinylated C-terminal avidin tag (**Fig. 1A**, right). Steady-state SPR provided a K_D_ of 5.8 µM, consistent with ITC. Slightly higher K_D_ values obtained from kinetic analysis (K_D_=16.2 µM) are likely due to non-optimal curve fitting and fast kinetics. The observed characteristic block-shaped kinetic profile with extremely fast on- and off-rates is indicative of rapid binding and release of suramin by ISG75.

### CRISPR-Cas9 disruption the ISG75 locus

**Figure 2:**
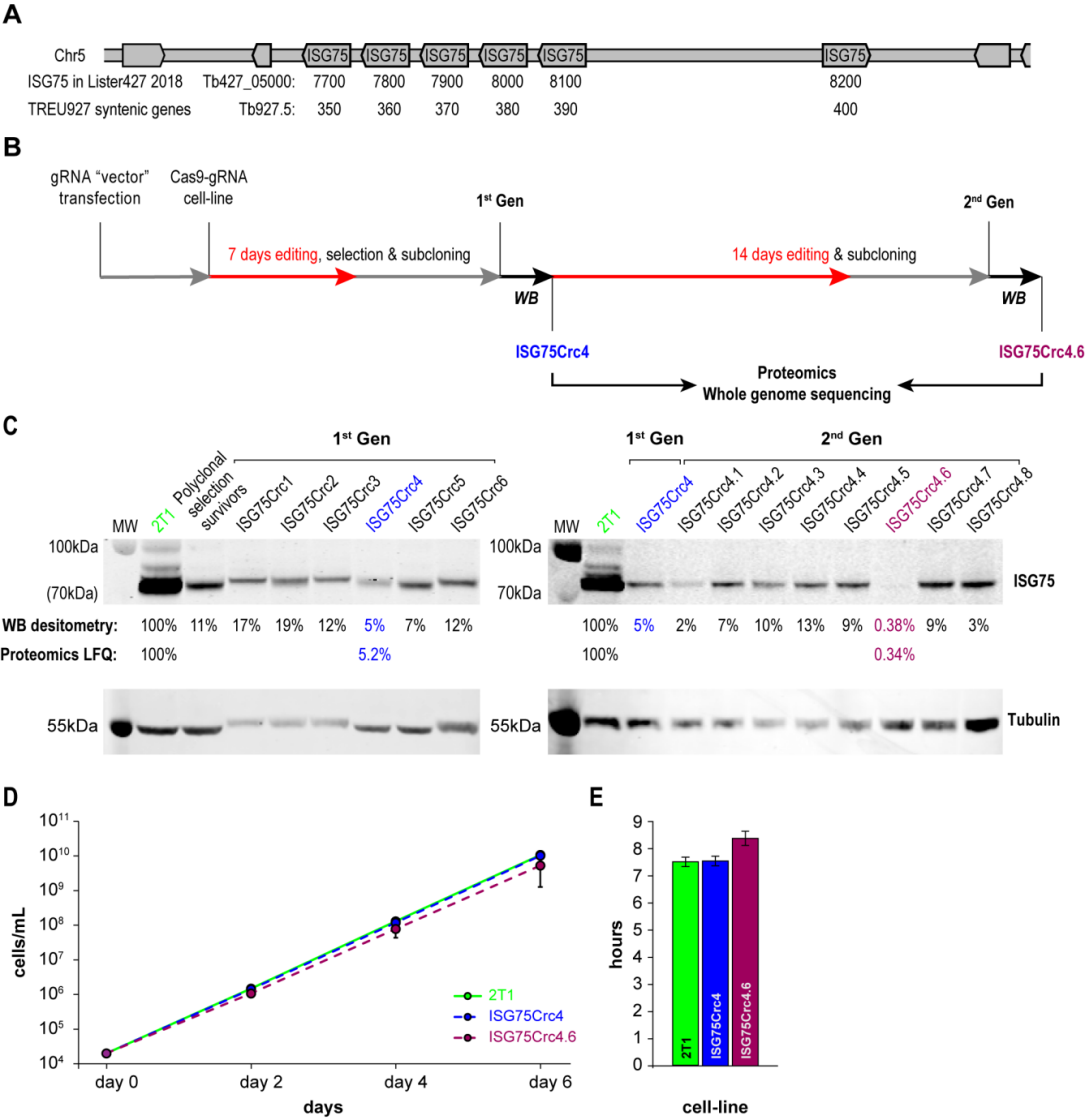
FIGURE 2: CRISPR-Cas9 strategy for obtaining ISG75 knockout. **(A)** ISG75 locus map in *T. brucei* Lister 427. **(B)** Following delivery and selection for cells stably expressing a chosen gRNA, Cas9 was induced for 7 days and the population selected with suramin (70µm) for 4 days, then subcloned and individual clones subjected to Western blot analysis (WB) (in **C**). Clone 4 termed ISG75Crc4 and expressing ∼5% of ISG75 parental levels was subjected to an additional 14 days of Cas9 induction, subcloned again and individual clones again subjected to Western blot analysis. Clones ISG75Crc4 and ISG75Crc4.6 were analysed by whole genome sequencing and proteomics to assess respectively the genomic architecture of the edited ISG75 locus and remaining protein levels. **(C)** Western blot and proteomics analysis of first- and second-generation clones reveals ISG75Crc4 and ISG75Crc4.6 as 95% knockdown and effective knockout clones respectively. Numbers given are Western blot densitometry values for 1^st^ and 2^nd^-generation clones and proteomics label free quantification for clones ISG75Crc4 and ISG75Crc4.6. **(D)** Cumulative growth curves over six days of cultivation for parental 2T1 (green), mutant ISG75Crc4 (blue) and ISG75Crc4.6 (purple) cell-lines. Three replicates for each line were grown in parallel. Error bars, standard deviations. **(E)** Average doubling times for 2T1, ISG75Crc4 and ISG75Crc4.6, calculated from **(D)**.

A modest two-fold increase in EC_50_ was observed from ISG75 knockdown (from 18 nM to 39 nM [17]), but as these cells retain a low level of ISG75 expression, it was unclear if this was due to residual receptor activity or indicative of an additional pathway. ISG75 is encoded by an array of six genes on chromosome V from Tb427_050007700 to 050008200 in *T. brucei* Lister strain 427 2018, TriTryp. For the purposes of editing and proteomics in this work we use gene IDs and sequences from the re-sequenced *T. brucei* Lister strain 427 [46], that identified five contiguous open reading frames on one strand (Tb427_050007700, 7800, 790, 8000 and 8100) and one on the opposite strand (Tb427_050008200; **Fig. 2A**). For the purposes of clarity we also give equivalent Tb927 gene IDs Tb927.5.350, 5.360, 5.370, 5.380, 5.390 and 5.400 that compose a syntenic locus of six genes in *T. brucei* TREU927. The majority of these genes encode near identical copies at the nucleotide level, with a more variant copy, Tb427_050007700, at one end of the array (Suppl. Fig. S2). To achieve complete depletion, we employed CRISPR-Cas9 based on delivery and genomic incorporation of a cassette consistently expressing gRNA into a cell line inducibly expressing Cas9 [47, 48]. To avoid designing a gRNA to each gene we employed GPP sgRNA designer (https://portals.broadinstitute.org/gpp/public/analysis-tools/sgrna-design) I [49, 50] to rank gRNA against a target sequence with maximal on-/minimal off-target activity. Since genes encoding ISG75 are nonidentical, only gRNA sequences in conserved regions were considered and, since GPP sgRNA designer does not include kinetoplastid genomes, candidate gRNAs were ranked by their on-target activity, then blasted against the *T. brucei* Lister 427 and TREU927 genomes and off-target effects estimated using the Rule Set II substitutions matrix [49]. We gave preference to gRNAs targeting the 5' end of the gene to minimise potential of expression of ISG protein fragments of significant size.

The ISG75 locus is suitable for CRISPR-Cas9 editing, as it possesses multiple NGG (any-guanine-guanine) protospacer adjacent motif (PAM) sequences recognisable by Cas9 within conserved regions. More importantly, ISG75 depletion bolsters the suramin EC_50_ to above the EC_100_, facilitating selection with suramin [17]. A sequence spanning nucleotides 34 to 53 was selected for CRISPR-Cas9 targeting (Fig. S2) and the corresponding gRNA expression cassette introduced into the p2T1-T7Cas9 cell line [48]. Transformed cells were selected with phleomycin and Cas9 expression induced with tetracycline (Tet) in the surviving polyclonal cell population. Tet induction in the 2T1-T7Cas9 can achieve complete CRISPR-driven disruption of a single gene locus in two to three days [48], but to disrupt the multi-gene ISG75 locus induction was initially for seven days (**Fig. 2B**). The polyclonal CRISPR-edited cell population was selected with 70 nM suramin for four days and subcloned (first-generation clones). The length of suramin selection is sufficiently short to minimise the possibility of suramin resistance mutations unrelated to ISG75, e.g. selection for VSG^sur^ [38]. Note, that while a direct ortholog of VSG^sur^ in *T. brucei* Lister 427 or *T. brucei brucei TREU927* has not been identified, the resulting edited lines were clearly not expressing a VSG^sur^ as evidenced by the absence of a comparable increase in suramin resistance (see below).

The level of ISG75 decreased to 11% in the suramin survivor population, and individual clones varied between 5% and ∼20%, as monitored by Western blotting (**Fig. 2C**), suggesting that at least one ISG75 gene remained intact. In pursuit of a complete knockout, first-generation ISG75Crc4 cells with 5% ISG75 expression, were subjected to 14 additional days of induction; subsequent subcloning yielded an effective knockout clone ISG75Crc4.6, with less than a half of one percent ISG75 expression estimated by Western blot (**Fig. 2C**). The ISG75 antibody used here was raised against a long peptide fragment from one ISG75 paralog and never formally proven to recognise all ISG75 proteins; hence we validated ISG75Crc4 and ISG75Crc4.6 knockouts by both genome resequencing and whole-cell proteomics.

Whole-cell proteomics confirmed the Western blot analysis. Total ISG75-derived peptides were decreased by ∼95% as judged by LFQ intensity in ISG75Crc4, but residual peptide levels were detected for five of six ISG75 paralogs across multiple replicates (**Fig. 2C**, S3A and Table S1). Interestingly, peptides for the sixth gene product, Tb427_050007700, were only found in one of five parental 2T1 replicates and undetectable in ISG75Crc4, indicating inherently low expression for this paralog (Fig. S3A and Table S1). Concurrent with proteomics data, whole genome sequencing (WGS) subsequently revealed localised disruption of the ISG75 locus with noticeable, albeit as expected, incomplete read depletion for the five highly expressing genes (Supplementary Results, Fig. S3A and S4).

In ISG75Crc4.6 cell lysates peptides were detected only for a single ISG75 paralog Tb427_050007900, and overall ISG75 levels decreased by >99% by LFQ intensity (**Fig. 2C**, Fig. S3A and Table S1). WGS revealed gaps in coverage and, thus, functional gene deletion across the majority of the locus (Supplementary Results, Fig. S3A and S4) – concurrent with both Western and proteomics analysis. WGS additionally revealed that ISG75Crc4.6 has a portion of chromosome V deleted and thus is over-edited (Fig. S3B). Despite this, we note that ISGCrc4.6 displayed no obvious phenotype besides a slight increase in doubling time to 8.4 hrs from 7.6 hrs in both parental 2T1 and ISG75Crc4 cell-lines (**Fig. 2 D** and **E**), and a small tentative accumulation of 1N2K phase cells (Fig. S5). Further, of the 27 genes (and pseudo-genes) contained in the deleted portion of chromosome V in ISG75Crc4.6 (Table S1, deleted genes) none were previously identified by the RIT-seq screen as involved with suramin or as essential [17]. We thus considered ISG75Crc4 and ISG75Crc4.6 as respective 95% knockdown and complete knockout mutants suitable for suramin resistance assessment and further analysis.

### ISG75 knockout has moderate impact on suramin sensitivity

**Figure 3:**
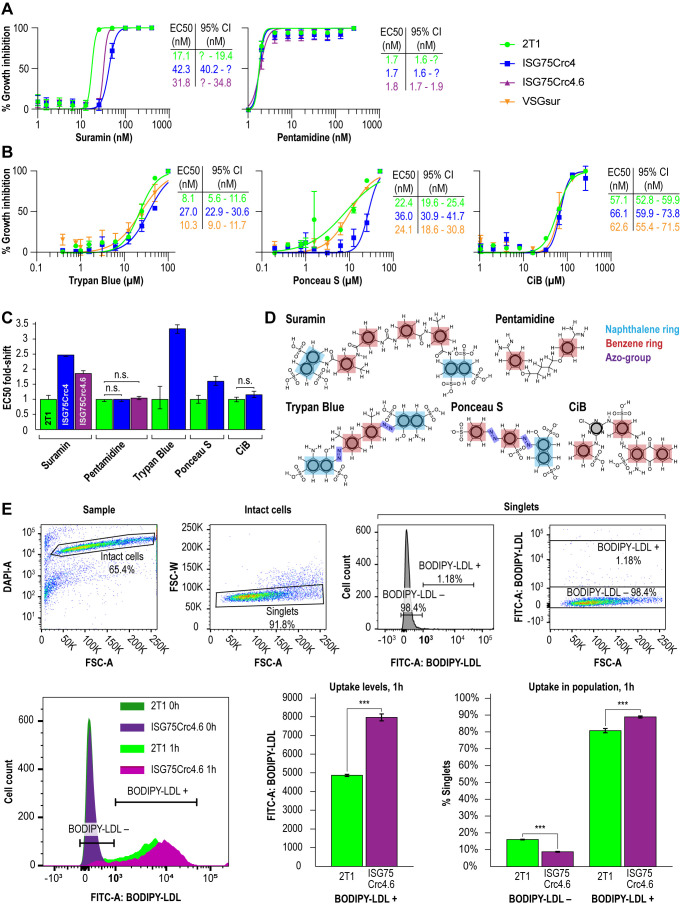
FIGURE 3: Impact of ISG75 on drug sensitivity. **(A)** EC_50_ curves showing shift in resistance to suramin and pentamidine in ISG75Crc4 and ISG75Crc4.6 cell-lines. **(B)** EC_50_ curves showing shift in resistance to trypan blue, ponceau S and cibacron blue in ISG75Crc4 compared to VSG^sur^. **(C)** Dynamite-plot showing respective shifts in EC_50_. Error bars, 95% confidence interval (CI). Asymmetric confidence intervals (95% CI) were calculated where possible in GraphPad PRISM and statistical significance of each EC_50_ shift assessed by confidence interval overlap – overlapping CI indicated absence of statistically significant shift. **(D)** Structures of drugs studied. Highlighted in light blue are naphthol moieties in suramin, trypan blue and ponceau S in red are single aromatic benzene rings and in dark blue are azo-groups. **(E)** Flow cytometry gating strategy (top) and representative flow cytometry histogram with bar-plots (bottom) showing LDL uptake upon knockout of ISG75 in ISG75Crc4.6. Bar plot error bars, standard errors from biological replicas, *** indicates p-value < 0.01.

Direct binding of suramin by ISG75 suggests receptor-mediated endocytosis as the mechanism. Both ISG75Crc4 and ISG75Crc4.6 cells express lower levels of ISG75 than RNAi-silenced cells, however the EC_50_ of both ISG75Crc4 and ISG75Crc4.6 are comparable: we recorded EC_50_ of 42 nM for ISG75Crc4 and 31 nM for ISG75Crc4.6 versus that of 39 nM for silenced cells when cultured under identical conditions [17] (**Fig. 3A** and **C**). By comparison, the EC_50_ for pentamidine was unchanged in these lines. In view of a 100-fold increase in suramin EC_50_ in cells which express VSG^sur^ [38], this smaller ΔEC_50_ for ISG75-depleted cells suggests the presence of an ISG75-independent route for suramin entry.

In line with direct interactions between trypan blue and ISG75, we further assessed changes in trypan blue sensitivity as well as the structurally related multi-naphthalene ring compounds Ponceau S and Cibacron blue. The latter is polysulphonated but lacks a naphthol moiety (**Fig. 3B**). ISG75Crc4 exhibited similarly small increases in EC_50_ for trypan blue and Ponceau S, 3.4- and 2.8-fold respectively, while the EC_50_ for Cibacron blue remained unchanged. By comparison, the VSG^sur^ cell-line exhibited no notable shift in sensitivity to these compounds and highlights the specific role of ISG75 and suggests that the naphthol group is important for binding (**Fig. 3B-D**).

### ISG75 is not the LDL receptor

While suramin uptake correlates with serum levels of LDL, LDL uptake itself is abrogated by suramin [39]. This suggests a potential competition for receptor binding sites between suramin and LDL or that suramin intoxication leads to a defect in the LDL uptake pathway. Notably, the LDL receptor remains unidentified [40, 51]. Therefore, to determine if LDL uptake can occur in the ISG75 knockout cells we assayed LDL accumulation using flow cytometry. LDL uptake appeared increased in ISG75Crc4.6 (**Fig. 3E**); the absence of any decrease clearly indicates that ISG75 is not required for LDL uptake.

### ISG75 depletion does not change the effects of suramin on the proteome

The moderate increase in suramin resistance in ISG75 silenced or knockout trypanosomes suggests additional routes of entry for suramin. We asked whether ISG75-dependent uptake has a specific impact on the effects of suramin on cells, i.e., if there is a signalling mechanism associated with ISG75-mediated endocytosis of suramin. Metabolomics and proteomics have indicated specific metabolic reprogramming and activation of mitochondrial pathways, plus upregulation of differentiation/stress-associated genes, following suramin exposure [37].

**Figure 4:**
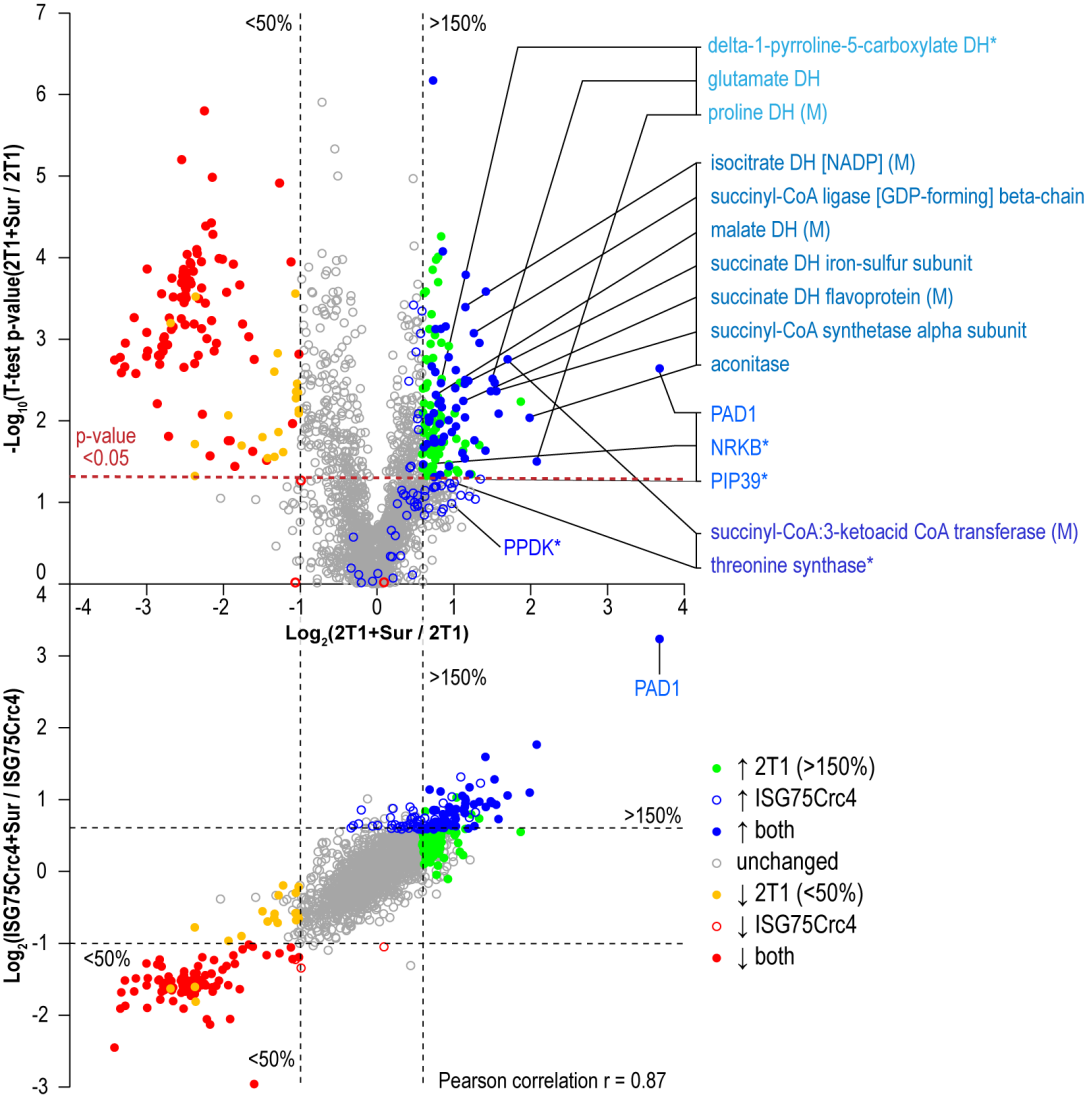
FIGURE 4: Changes in protein expression levels in suramin-treated cells. Volcano plot of protein level changes in parental cells with and without suramin (top). Coloured respectively are proteins significantly up- or down-regulated (p-value < 0.05) only in parental, only in ISG75Crc4 and in both as indicated after exposure to suramin. Additionally highlighted in coloured groups (top to bottom, lighter to darker blue shades) are proteins involved in proline catabolism, mitochondrial activation and Krebs' cycle, early BSF/PCF differentiation markers, and proteins involved in threonine metabolism as indicated. Single asterisk (*) marks PIP39 and PPDK that display similar upregulation as shown previously, but did not reach statistical significance (p-values of 0.052 and 0.103 respectively); and NRKB and threonine synthase that displayed similar trends in parental and ISG75Crc4, but have low significance levels in one or both samples (NRKB, 1.5-fold increase in both parental and ISG75Crc4 with p-values of 0.034 and 0.093 respectively; TS, 1.9-fold increase with p-value 0.059 in parental and 1.7-fold increase with p-value 0.094 in ISG75Crc4). Correlation plot of proteins increased or decreased in parental and ISG75Crc4 cell-lines (bottom). Coloured dots are significantly altered proteins. Overall Pearson correlation coefficient r = 0.87.

Parental 2T1 and ISG75Crc4 cells were exposed to suramin at double their respective EC_50_ concentrations for 48 h and compared to respective untreated controls in whole-cell comparative proteomics analysis (2T1 v 2T1 + 2xEC_50_2T1 and ISG75Crc4 v ISG75Crc4 + 2xEC_50_ISG75Crc4). Collectively 3746 protein groups were identified by LC-MSMS, representing ∼40% of the total proteome. Of these, 1901 protein groups were found in at least two replicates for each condition (treated/untreated) and cell-line (2T1/ISG75Crc4). A total of 1282 protein groups were identified across every replicate (four replicates for 2T1 and three for each 2T1 + 2xEC_50_2T1, ISG75Crc4 and ISG75Crc4 + 2xEC_50_ISG75Crc4; Table S2). Suramin-induced changes were similar in parental and ISG75Crc4 cells (**Fig. 4**, top) and closely mirrored previous data [37]. Changes include upregulation of pyruvate phosphate dikinase (PPDK), enzymes of the glycolytic pathway and at least seven Krebs' cycle enzymes (Table S2). Whilst the magnitudes for proteome changes were not always identical to earlier work, the overall changes were very similar, and we suggest that any differences reside within the experimental protocol and technical variability. Further mirroring previous data, the early differentiation/stress markers PAD1, PIP39 and NRKB were upregulated to similar levels in parental and ISG75Crc4 cells (Table S2). Significantly, the sole example of a protein with altered expression upon suramin treatment that was separately affected by the loss of ISG75 alone was succinyl-CoA:3-ketoacid coenzyme A transferase (Tb927.11.2690): its protein levels stood at 140% of the parental 2T1 levels in ISG75Crc4, and this likely resulted in lower relative upregulation upon suramin treatment in the ISG75 depleted cell line, i.e. two-fold versus 3.2-fold in 2T1. The correlation between proteome changes in parental and ISG75Crc4 cells upon suramin treatment is 0.87 (Pearson coefficient; **Fig. 4**, bottom), indicating high concordance. Hence, we conclude that suramin-induced increases in glycosomal and mitochondrial ATP production and differentiation/stress marker expression are independent of ISG75 expression.

### ISG75 interacts with protein expression and Ca^2+^-sensing pathways

**Figure 5:**
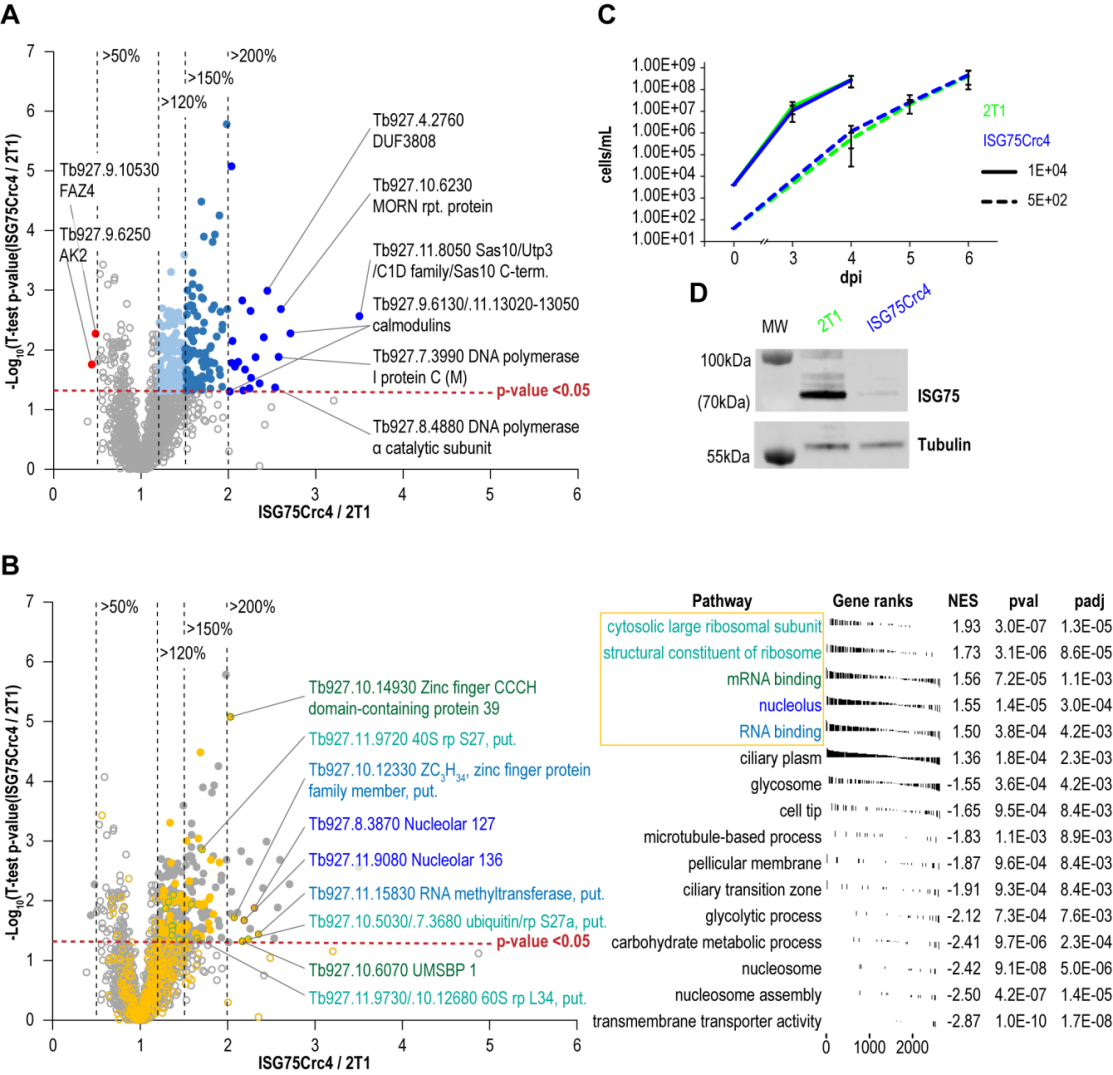
FIGURE 5: Changes in protein expression levels in ISG75 knockout cells. **(A)** Volcano plot showing distribution of protein level changes in in ISG75Crc4 cells. Proteins and protein groups increased 1.2-fold, 1.5-fold to 2-fold and 2-fold or more with p-values < 0.05 are shown respectively in light blue, turquoise and dark blue filled circles, protein groups decreased 2-fold or more - in red, non-significantly shifting protein groups – in grey unfilled circles. **(B)** FGSEA output showing most enriched GO-terms among all proteins in A. Top five groups are shown in orange. Unfilled circles all proteins, filled circles proteins with increased expression **(Table 1** and **2)**. Labelled circles with coloured outlines are proteins with most increased expression in these groups. **(C)** Parasitaemia levels in Balb/C mice blood on days three and four post infection with 10^4^ ISG75Crc4 cells and days four to six post infection with 10^2^ cells, top and bottom respectively. Error bars, standard error. **(D)** Western Blot analysis of lysates of 2T1 and ISG75Crc4 cells extracted on day four terminal bleeds of mice infected with 10^4^ cells shows persistent depletion of ISG75 in trypanosomal cell when in host environment – similar to that observed *in vitro*.

To further assess cell-wide changes, parental and ISG75Crc4 trypanosomes were subjected to proteome analysis at greater depth: five replicates, each divided into three slices, were analysed for parental and ISG75Crc4 cell lines (**Fig. 5**, Table S1). From this, 4625 protein groups were identified, including 24 groups representing the ISG superfamily and matching the expected depth for whole-cell proteomics [2, 37]. A total of 2145 protein groups were robustly identified in all five replicates in both parental and ISG75Crc4 and a further 778 protein groups in three or more replicates in both cell lines (excluding ISG protein groups). Only 21 proteins/protein groups were significantly increased (p-value <0.05) between two and 3.5-fold in ISG75Crc4 with a further 99 with modest >1.5-fold increases (**Fig. 5A**). Only two proteins were decreased to below 0.5-fold of parental cell level.

FGSEA (Fast pre-ranked Gene Set Enrichment Analysis) GO-term analysis of the 2145 reliably identified proteins revealed five overrepresented categories; cytosolic large ribosomal subunit (GO:0022625), structural constituent of ribosome (GO:0003735), mRNA binding (GO:0003729), nucleolus (GO:0005730) and RNA binding (GO:0003723) (**Fig. 5B**). These five GO-term groups are functionally related and encompass 36 out of 120 significantly increased proteins (1.5-fold and above; **Table 1**), providing evidence for increased nucleolar and ribosomal activity and post-transcriptional regulation of gene expression: the 1.2- to 2.2-fold increase in levels of fourteen 40S and 60S ribosomal proteins that are already highly abundant is significant. Further, Tb927.11.8050, the most upregulated protein at 3.5-fold, contains Utp3 and Sas10 domains commonly found in the U3 ribonucleoprotein complex [52, 53], and Tb927.8.4880, a DNA polymerase I subunit is increased 2.5-fold. Upregulation of these two proteins further supports increased ribosomal activity.

**Table 1.** **List of 1.5-fold or higher proteins with increased expression (p-value <0.05) in the top five most enriched groups in the FGSEA analysis in ISG75Crc4 cells.** Given are enriched GO-terms, gene IDs and annotations along with fold increase. Additionally listed related proteins among the topmost proteins with increased expression in ISG75Crc4.

**TriTrypDB accession**	**Product Description**	**GO:0022625** cytosolic large ribosomal subunit	**GO:0003735** structural constituent of ribosome	**GO:0003729** mRNA binding	**GO:0005730** nucleolus	**GO:0003723** RNA binding	**Fold change**
Tb927.10.5030; Tb927.7.3680	ubiquitin/ribosomal protein S27a, putative; ubiquitin/ribosomal protein S27a, putative						2.2
Tb927.11.9720	40S ribosomal protein S27, putative		+			+	1.7
Tb927.11.9730; Tb927.10.12680	60S ribosomal protein L34, putative; 60S ribosomal protein L34, putative	+	+		+		1.7
Tb927.9.15170; Tb927.9.15110	60S ribosomal protein L5, putative; 60S ribosomal protein L5, putative	+	+		+	+	1.6
Tb927.6.5130; Tb927.6.5120	60S acidic ribosomal protein P2, putative; 60S acidic ribosomal protein P2, putative	+	+				1.5
Tb927.10.6070	universal minicircle sequence binding protein 1			+	+		2.2
Tb927.10.14930	Zinc finger CCCH domain-containing protein 39			+		+	2.0
Tb927.11.6440	hypothetical protein, conserved			+			1.9
Tb927.1.2600	pumilio/PUF RNA binding protein 9			+		+	1.8
Tb927.7.2170	Nucleolar protein 168			+	+		1.8
Tb927.11.14750	PSP1 C-terminal conserved region, putative			+	+		1.8
Tb927.11.6740	pumilio/PUF RNA binding protein 10, putative			+	+	+	1.8
Tb927.11.6430	hypothetical protein, conserved			+			1.8
Tb927.3.3940; Tb927.3.3960; Tb927.3.3930	RNA-binding protein; RNA-binding protein, putative; Double RNA binding domain protein 6A			+		+	1.7
Tb927.6.4440	RNA-binding protein 42 (RNA-binding motif protein 42)			+			1.7
Tb927.4.2030	DNA/RNA-binding protein Alba 4			+	+	+	1.7
Tb927.1.4690	Protein arginine N-methyltransferase 1 catalytic subunit			+	+		1.5
Tb927.6.5010	hypothetical protein, conserved			+			1.5
Tb927.8.3870; Tb11.v5.0681.1	Nucleolar protein 127				+		2.3
Tb927.11.9080	Nucleolar protein 136				+		2.2
Tb927.11.8270	hypothetical protein, conserved				+		1.8
Tb927.10.13310	DNA-directed RNA polymerase I subunit RPB5z, putative				+		1.7
Tb927.10.15270; Tb11.v5.0998.1; Tb11.1240	methionine aminopeptidase, type I, putative; metallo-peptidase, Clan MG, Family M24				+		1.7
Tb927.4.4950	Nucleolar protein 61				+		1.7
Tb927.8.1980	Beta propeller protein 1				+		1.7
Tb927.6.1900	essential nuclear protein 1, putative				+		1.6
Tb927.9.6940	DNA topoisomerase type IB small subunit				+		1.5
Tb927.10.14680	ribosome biogenesis protein, putative				+		1.5
Tb927.2.5130	hypothetical protein, conserved				+		1.5
Tb927.8.5880	eukaryotic translation initiation factor 1A, putative				+	+	1.5
Tb927.7.5490	protein arginine n-methyltransferase 7				+		1.5
Tb927.11.5250	hypothetical protein, conserved				+		1.5
Tb927.11.15830	RNA methyltransferase, putative					+	2.4
Tb927.10.12330	zinc finger protein family member, putative					+	2.1
Tb927.11.2370	mRNA export factor MEX67					+	1.6
Tb927.9.3760	Nucleoporin GLE2					+	1.5
Tb927.11.8050	Sas10/Utp3/C1D family/Sas10 C-terminal domain containing protein, putative	3.5					
Tb927.8.4880	DNA polymerase alpha catalytic subunit	2.5					

A sixth cohort, related to ciliary plasm (GO:0097014), was unexpected and includes calmodulins (Tb327.9.6130, increased 2.7-fold and Tb927.11.13020-Tb927.11.13050, increased 2.0-fold), suggesting Ca^2+^ signalling interactions. A MORN protein, Tb927.10.6230, was also considerably increased in expression by 2.6-fold and multiple flagellar components are modestly upregulated (**Table 2**). Together with a decrease of 50% in FAZ4 (Tb927.9.10530), these data suggest a potential involvement of ISG75 in Ca^2+^ signalling, with possible connections to flagellar organisation and may indicate interactions with, for example, adenylate cyclases [54].

**Table 2.** **List of cilia (6^th^ top FGSEA cohort) and Ca+ sensing proteins increased or decreased in ISG75Crc4.** Given are rank up-regulation, gene ID, database annotation and fold upregulation.

**Rank**	**TriTrypDB accession**	**Product Description**	**Fold change**
2	Tb927.9.6130	calmodulin, putative	2.7
3	Tb927.10.6230	MORN repeat containing protein	2.6
8	Tb927.11.15830; Tb11.v5.0683.1	RNA methyltransferase, putative	2.4
21	Tb927.11.13050; Tb927.11.13040 Tb927.11.13030; Tb927.11.13020	calmodulin	2.0
49	Tb927.9.10370	TAX-1	1.7
162	Tb927.11.3360	Component of motile flagella 22	1.4
171	Tb927.11.12150	flagellar pocket cytoskeletal protein bilbo1	1.4
221	Tb927.3.5020	Flagellar Member 6	1.3
234	Tb927.10.8940	flagellum targeting protein kharon1, putative	1.3
377	Tb927.9.10530	Flagellum attachment zone protein 4	0.5

### ISG75 depletion has no effect on the level of proteins implicated in ISG-associated internalization, turnover, and ER quality

Internalisation and turnover of ISGs in trypanosomes has been shown to depend on the ESCRT/ubiquitylation system [7, 8, 55, 56] and two deubiquitylating enzymes (DUBs) TbVdu1 and TbUsp7 [57]. Notably, neither DUB was affected by ISG75 depletion, and concomitant with steady levels of TbUsp7, we noted no effect on the levels of TbSkpZ (Tb927.10.11610), an adapter in the trypanosome-specific TUS deubiquitinase complex consisting of TbSkpZ, TbTpr86 and TbUsp7 proteins [58]. We additionally found no change in levels of several components of ESCRT machinery that partake in turnover of the related invariant surface glycoprotein ISG65, such as TbRab7 (Tb927.9.11000), TbVps4 (Tb927.3.3280) [56], TbVps24 (Tb927.11.10000) [59], TbRab11 (Tb927.8.4330) [60], TbFab1 (Tb927.11.1460) [61] and TbRab28 (Tb927.6.3040) [62]. Finally, we considered potential relationships between ISGs and the ER quality control/folding system [63]. Only modest alterations were observed for any of these proteins, and we conclude there is no evidence for a substantial impact to biosynthetic or endocytic processes by ISG75 depletion (Table S1).

### ISG75 depletion is non-critical for infection and proliferation in a small animal model

We finally asked if ISG75 contributes towards *T. brucei* bloodstream form proliferation *in vivo*. We infected Balb/C mice with ISG75Crc4 and parental cells; proliferation of both cell lines were essentially indistinguishable when infecting with either or 10^2^ or 10^4^ cells [64] (**Fig. 5C**). Western blot analysis confirmed that ISG75Crc4 cells following cultivation in mice were expressing ISG75 at sub 5% levels, consistent with *in vitro* conditions (**Fig. 5D**). ISG75Crc4.6 was not used in mouse experiments as any of the effects would have been uninterpretable due to the genes additionally deleted from this cell-line. With the caveat that the Lister 427 strain is highly virulent, these data suggest that ISG75 is not critically involved in bloodstream form survival and proliferation.

## DISCUSSION

The interaction between infectious agents and drugs is frequently complex, which can explain both specificity and high potency [65]; insights into these relationships are of notable value for drug discovery and understanding resistance. Suramin is an old drug with a complex mode of action and, despite recent advances, a complete description of the impact against trypanosomes remains to be achieved. Suramin likely exploits ISG75 as an uptake route and elicits a rapid collapse in intracellular ATP [37], but several questions remained. Firstly, it was unclear if suramin interacts directly with ISG75 and, considering the clear prominence of ISG75 on the trypanosome surface, if ISG75 represented the sole route for entry into the trypanosome. Secondly, if an apparent link between suramin and LDL uptake represented an ISG75 independent route. Thirdly, the ISG75 contribution to signaling roles and overall trypanosome fitness was not known.

ISG75, suramin and trypan blue directly interact by a hydrophobic mechanism, with two suramin molecules binding each ISG75 monomer. There is no obvious sequence repeat within ISG75, suggesting that the two suramin binding sites are non-identical and independent of an oligomeric state, unlike interactions between the trypanosome transferrin receptor and transferrin [45]. ISG75 depletion enhances resistance to suramin, trypan blue and Ponceau S, all of which contain naphthalene aromatic ring substituents. By contrast, sensitivity to both cibacron blue and pentamidine, that contain only phenyl moieties, was unaffected by ISG75 knockdown, suggesting that the naphthalene determinant is essential for ISG75 binding. As the extracellular domain of ISG75 carries a net negative charge at neutral pH, this is consistent with ionic interactions being thermodynamically unfavourable and hydrophobic interactions dominating the binding energy.

In generating the ISG75 knockout we observed over-editing, likely due to a prolonged editing period. The ISG75Crc4.6 line lost a ∼100kb region of chromosome V adjacent to the ISG75 locus and almost certainly resulting from multiple double-strand breaks introduced by Cas9. The deletion has little impact on fitness, at least for the functions analysed here as well as the viability of the clone, but highlights the importance of full validation of edited lines. While the intended ISG75 deletion is detectable by simple PCR to validate removal of genes of interest, additional off-target genome changes require genome resequencing.

In treating trypanosomiasis a dose of ∼150 μM suramin is administered, but ∼80% of the drug is sequestered by LDL and other serum components [39, 43], but a free suramin bloodstream concentration of ∼15 μM is sufficient to utilize a receptor with moderate affinity, such as ISG75 with a K_D_ ∼3 μM. The kinetics of the suramin-ISG75 interaction have a catch-release profile, allowing rapid suramin uptake in the presence of free suramin, while the high off-rate with a complex half-time of ∼5 seconds allows the ligand to dissociate rapidly inside the trypanosome endosomes, where there is low free suramin, providing a mechanism to concentrate suramin. Evidence for ISG75-dependent suramin uptake is supported by at least four lines of evidence: The increase in EC_50_ following ISG75 silencing [17], a similar ΔEC_50_ observed in ISG75 nulls described here, correlation between ISG75 levels and suramin uptake [37] and direct binding of suramin by ISG75 at physiologically relevant concentrations. However, the small suramin ΔEC_50_ following ISG75 knockdown/knockout is distinct from the 100-fold change in suramin resistance associated with expression of VSG^sur^, indicating that much greater resistance to suramin can arise. VSG^sur^, present at ∼1000-fold greater copy number than ISG75, likely acts simply as a sink for suramin, lowering the concentration below the therapeutic level [42].

Further, knockdown of MFST, the lysosomal suramin transporter, leads to a ten-fold ΔEC_50_ and also suggests that additional, ISG75-independent routes for suramin uptake exist. Suramin accumulation correlates with levels of both LDL and albumin in serum [38, 39], but multiple lines of evidence point away from ISG75 acting as an LDL receptor, including altered LDL uptake by VSG^sur^ expressing cells [38], endocytosis mutants affecting LDL uptake but showing no impact to suramin EC_50_ [41], and, as shown here, that LDL uptake is not abrogated by ISG75 knockout (**Fig. 3**). Lastly, our data indicates that ISG75 depletion does not alter cellular responses to suramin exposure at the protein level. Taking this evidence in consideration, we propose that at least three mechanisms can contribute towards suramin resistance: Identity of the specific VSG, ISG75 as a receptor and a third mechanism that may depend on serum components.

While subtle effects may pass undetected in our animal model, specifically due to immune system defects in Balb/c mice [66] and rapid proliferation of the Lister 427 trypanosome strain, ISG75 is clearly non-essential in culture and 95% depletion had no obvious impact on mammalian infection. While this does not exclude specific and important interactions with the host, and likely soluble serum factors as seen for many additional members of the VSG surface protein superfamily [5, 67–70], this is evidence of a more nuanced, rather than central or fundamental, role and surprising for a high abundance protein on the trypanosome cell surface. While the origins of VSG-fold containing proteins remain unclear, their multiple contributions to innate and acquired immune evasion in African trypanosomes represent a significant contribution towards exploitation of the mammalian host.

Finally, the ISG75 knockout cell line exhibits significant changes at the proteome level, for example upregulation of multiple ribosomal components and calmodulins. This suggests that while there is no gross impact to fitness, the parasite does react to loss of a major surface trans-membrane domain protein, and which may have activated translational mechanisms together with possible signalling pathway components. Together with evidence for involvement in Ca^2+^ signalling, adenylate cyclase activity and possible flagellum interactions suggests a role for ISG75 in control of proliferation, albeit one not detected here where culture conditions are highly permissive.

## MATERIALS AND METHODS

### Expression and purification of untagged ISG75 for ITC

The gene fragment coding for the extracellular domain (residues 29-462) of TbgISG75 (*T. b. gambiense* LiTat 1.3, accession number DQ200220.1) was cloned into the pExpress2.1 vector (Expres2ion Biotechnologies). Recombinant ISG75 comprising an N-terminal secretion signal and a C-terminal HRV3C-cleavable C-tag was produced in *Drosophila melanogaster* S2 cells following manufacturer's recommendations (Expres2ion Biotechnologies). Briefly, 5mL of 2x10^6^ cells/mL, cultured in Ex-cell 420 Insect serum free medium (14420C, SAFC Biosciences) supplemented with Pen/Strep solution, were transfected with 0.25 mL of lipofectamine-based reagent (Expres2ion Biotechnologies) and 12.5 μg plasmid DNA. After 4 hours, 12.5% FBS was added, and cells were incubated in T25 flasks overnight. After 24 hours, selection was initiated by adding 2 mg/mL zeocin to generate a stable ISG75-expressing cell line. After 3-4 weeks of selection cells were transferred to shaker flasks and production scaled up. Cells were harvested after 4 days of expression by centrifugation at 1000*g* at 15°C for 15 minutes. ISG75-containing supernatant was subsequently centrifuged at 13000*g* at 15°C for 10 minutes and filtered through 0.22 μm filter. The cleared supernatant was concentrated and exchanged into buffer A (20 mM Tris pH 7.5, 150 mM NaCl), using tangential flow filtration. The protein solution was loaded onto 5 mL of column-packed CaptureSelect C-tag Affinity Matrix (ThermoFischer) using AKTA Start. After washing with buffer A, C-tagged ISG75 was eluted with buffer B (buffer A + 2 M MgCl_2_). To remove the C-tag the eluate was dialysed into buffer A in the presence of 200 μg of GST-tagged HRV3C protease. The protease was subsequently removed using a 1 mL GST Hitrap column and the flow-through fraction containing tag-free ISG75 was further purified by size exclusion chromatography on Superdex200 10/30 column (Supplemental data), equilibrated in buffer C (20 mM HEPES pH 7.5, 150 mM NaCl). Fractions containing monodisperse >95% pure ISG75 (Supplemental data) were pooled and flash-frozen in liquid N_2_.

### Biotinylated ISG75 for SPR

The ISG75 encoding gene fragment as above was also cloned into the mammalian expression vector pHLsec [71]. Recombinant ISG75 expressed from this vector sported an N-terminal mammalian secretion signal peptide and a C-terminal thrombin cleavage site, 6x-histidine and an Avi-tag. Transient expression was carried out in Expi293F cells, following the manufacturer's instructions (ThermoFisher). Briefly, 200 mL of cell culture (2.5 x10^6^ cells/mL) were transfected with 200 μg of plasmid DNA (in 5 mL of Opti-MEM medium), complexed with 534 μL ExpiFectamine^TM^ 293 Reagent (in 5 mL of Opti-MEM), in the presence of 5 μM kifunensine. 18 hours post-transfection, ExpiFectamine 293 Enhancers 1 and 2 were added and cells were cultured for an additional 48 hours. Cells were harvested at 300*g* at 4°C for 10 min. The ISG75-containing supernatant was filtered through a 0.22 μm membrane and dialysed into buffer D (20 mM Tris-HCl pH 8.0, 150 mM NaCl, 10 mM imidazole) at 4°C, overnight. ISG75 was affinity-purified using Ni-NTA Agarose (Qiagen) and eluted with buffer E (20 mM Tris-HCl pH8.0, 150 mM NaCl, 500 mM imidazole). For further purification the Ni-NTA eluate was injected onto a Superdex200 10/30, equilibrated in buffer C and fractions containing monodisperse ISG75 were pooled. To obtain biotinylated ISG75, 100 μL of ∼60 μM ISG75 were mixed with 2 μL of 5 mM biotin, 5 μL of 100 mM MgCl_2_, 2 μL of 100 mM ATP and 2 μL of 50 μM BirA ligase, incubated for 2 hours at 30°C. Molar equivalents of biotin, ATP and BirA ligase were added again for another 2 hours. GST-BirA ligase was removed by incubating the reaction mixture with 10 μL of Glutathione Sepharose 4 FF beads (Cytiva). Beads were removed by centrifugation and the reaction mixture dialysed into buffer C at 4°C, overnight. Biotinylation efficiency was analysed by a streptavidin shift assay. 10 μL aliquots of 28 μM biotinylated ISG75-his-Avi were flash-frozen in liquid N_2_ and stored at −80°C.

### Isothermal titration calorimetry

The thermodynamics of suramin/trypan blue ligand interactions with the extracellular domain of ISG75 were analysed by titration microcalorimetry using a MicroCal VP-ITC calorimeter (MicroCal, Malvern Panalytical Ltd). All experiments were performed in buffer F (100 mM Tris pH 7.5, 150 mM NaCl) at 25°C. The precise concentrations of ISG75, suramin and trypan blue were determined by quantitative amino acid analysis and elemental analysis, respectively. For suramin 2.1 mL of 16 μM ISG75 was titrated with 6 μL of 400 µM ligand per injection, while for trypan blue 15 μM protein and 500 µM ligand were used. Each of a total of 46 injections were 12 seconds long with 350 seconds spacing interval (500s for trypan blue) and 2 seconds filter period. In the control experiment, heat of dilution was measured by titrating ligand at a given concentration into 2.1 mL of buffer F, using the same parameters (Supplemental data). All titration data were processed using MicroCal Origin 7.0 software (MicroCal, Malvern Panalytical Ltd.)

### Surface plasmon resonance

Kinetics of suramin binding to the extracellular domain of ISG75 was determined by surface plasmon resonance (SPR) using a BIAcore T200 instrument (GE) and sensor chip CAPture (Cytiva). All experiments were performed in buffer G (20 mM HEPES pH 7.5, 150 mM NaCl, 3 mM EDTA, 0.005% (v/v) TWEEN-20). Biotinylated ISG75 stock (28 μM in buffer C) was diluted 40x in buffer G and immobilized to a series S sensor Chip CAP, at 10 μL/min for 120 seconds. ISG75 was then titrated with 1; 2; 4; 8; 16 and 32 μM suramin solutions prepared in buffer G, respectively, flowed at 30 μL/min for 300 s followed by 100 s dissociation time, using single capture method. The chip surface was regenerated between titrations with 6 M guanidinium hydrochloride dissolved in 0.25 M sodium hydroxide. Kinetic and steady state affinity parameters were evaluated using BIAcore T200 evaluation software (GE Healthcare).

### Genome editing and cell lines

The *T. brucei* Lister 427 2T1^T7Cas9^ cell-line [48], provided by David Horn, and its derivatives were cultured in HMI9 medium (Life Technologies), supplemented with 10% serum, blasticidin at 1 µg/mL, hygromycin at 1 µg/mL by default, and additional phleomycin at 2 µg/mL for gRNA cassette incorporation selection. 2T1^T7Cas9^:gRNA cell line was perpetually maintained with phleomycin 1 µg/mL. Cumulative proliferation was monitored by subculturing to 2 × 10^4^ cells/mL and counting cells bi-daily. Three replicates for each cell-line were grown in parallel over the period of six days. Doubling time was calculated as average of doubling times over this period.

ISG75 sequences for this study correspond to Tb427_050007700, Tb427_050007800, Tb427_050007900 Tb427_050008000, Tb427_050008100 and Tb427_050008200 from Lister strain 427 2018 assembly (TriTrypdb.org [46, 72]. Guide RNA against ISG75 was selected using GPP sgRNA designer (https://portals.broadinstitute.org/gpp/public/analysis-tools/sgrna-design) with Rule Set II [49, 50] and designed against Tb427_050007800 aligned with the remaining paralogs. Candidate gRNAs were ranked by on-target activity, checked for off-target effects against *T. brucei* Lister 427 and TREU 927 genomes using the RuleSet II substitutions matrix [49]. Selected gRNA targeted nucleotide sequence 34-53 nts (5'-GCAACAGTATTTCTCCTCTG-3') and was introduced into 2T1^T7Cas9^ cells as described [48]: Respective forward and reverse oligonucleotides 5'-AGGGGCAACAGTATTTCTCCTCTG-3' and 5'-AAACCAGAGGAGAAATACTGTTGC-3' were annealed by heating to 70°C and slow cooling and ligated into pT7^sgRNA^ vector linearized with BbsI. The construct was confirmed by sequencing, linearized with NotI, and gRNA cassette containing phleomycin resistance marker was used for electroporation into the 2T1^T7Cas9^ cell line. The parasite population was selected for cassette incorporation with phleomycin at 2 μg/mL for 7 days. Following successful cassette incorporation, gene editing was conducted by induction of Cas9 with tetracycline at 1 μg/mL for 7 days. Tetracycline was withdrawn and the culture further selected with 70 nM suramin prior to subcloning to obtain first generation clones. Selected clone 4 (ISG75Crc4) was further edited for 14 days as above, and the population subcloned to obtain second generation clones.

### Western blotting

Initial clone assessment was carried out by means of Western blot with rabbit anti-ISG75 (in house) at 1:2,000, anti-mouse b-tubulin (clone KMX-1; Millipore) at 1:10,000 used for normalisation. Following SDS-PAGE and transfer, membranes were blocked with TBS containing 5% (w/v) milk and 0.01% (v/v) Tween-20 (TBST, 1 hour) and probed with primary antibodies for 1 hour. Membranes were washed with TBS, exposed to secondary antibodies goat anti-rabbit IgG: IR Dye800RD and goat anti-mouse IgG: IRDye680CW (Li-COR) at 1:20,000 for 1 hour, then washed again with TBS. Imaging and quantitative signal detection was done using an Odyssey CLx Imager and Image-Studio software (Li-COR).

### Whole genome sequencing

Genomic DNA was extracted from parental, ISG75Crc4 and ISGCrc4.6 cells using QIAGEN Blood and Cell Culture DNA Kit (Cat. No. 13343) and sent to BGI Genomics (Hong Kong, China) for sequencing (100PE, HiSeq4000, PCR-free library). Reads were aligned to the Lister strain 427 2018 genome assembly using bowtie2 in very-sensitive mode and samtools-1.3 [73, 74] and visualized in the Artemis genome browser [75] for manual inspection of the genome and target ISG75 locus.

### EC_50_ determination

EC_50_ was determined using AlamarBlue in 96-well plates [76]. All drugs were titrated in two-fold dilutions: Suramin from 1.56 to 400 nM, Pentamidine from 0.98 to 250 nM, Trypan Blue from 0.2 to 50 nM, Ponceau S from 0.39 to 100 nM and CiB from 0.98 to 250 nM. Drug exposure was for 72 hours and AlamarBlue incubation overnight. Plates were read on an Infinite 200Pro plate-reader (Tecan) with the following parameters: Excitation 530nm; emission 585 nm; filter cut-off 570 nm. Data were analysed in GraphPad PRISM. Asymmetric confidence intervals (95% CI) were calculated where possible and statistical significance of each EC_50_ shift assessed by confidence interval overlap.

### LDL endocytosis

Parental and ISG75Crc4.6 were collected in log-phase by centrifugation at 2000*g* for 2 minutes in a swinging-tube rotor following by 5 minutes in a fixed-angle rotor, washed twice with HMI9 medium w/o serum, resuspended, and diluted to 2x10^6^ cells/mL. Cells were aliquoted as 285 µl in microfuge tubes and equilibrated at 37°C for 45 min prior addition of 15 µL of BODIPY^TM^-LDL (200 µg/mL) to a final concentration of 10 µg/mL. Control samples was immediately quenched by putting cells on ice and addition of 1 mL of ice-cold TDB buffer (5 mM KCl, 1 mM MgSO_4_, 22 mM (10:1) Na_2_H:NaH_2_PO4, 20 mM glucose, pH 7.8). Remaining samples were incubated at 37°C for 1 hour and quenched as above. Quenched samples were collected by centrifugation at 2,000*g*, 4°C for 5 minutes in pre-chilled swinging-tube rotor following by 5 minutes in a pre-chilled fixed-angle rotor, washed with 1.5 mL of ice-cold TDB, resuspended in 50 µL of TDB, and fixed for 5 minutes by addition of 50 µL of 10% (v/v) formaldehyde and DAPI to 1 µg/mL. Samples were washed by addition of 1400 µL TDB with 1% (w/v) BSA and 5 mM EDTA and centrifugation as above and resuspended in a final volume of 300 µL TDB, 5 mM EDTA. A total of 14 samples were prepared: in triplicate for parental and ISG75Crc4.6 at 0 h and 1 h; single replicas of parental at 1 h with unlabelled LDL or media alone. 10,000 cells from each sample were sorted on a FACS Canto flow cytometer (Becton Dickinson), using DIVA software. Forward scatter (FSC) and side scatter were detected from the 488 nm laser, BODIPY fluorescence detected at 530 nm using 488 nm excitation, and DAPI fluorescence at 450 nm using 405 nm excitation. Data analysis was performed using FlowJo (Treestar, Becton Dickinson). Gating was determined using a parental 0 h sample and propagated to all remaining samples. Intact cells were gated using FSC-A vs DAPI, singlets gated using FCS-A vs FCS-W. These were checked to capture correct populations across all samples. Gates for cells that did not take up any LDL (BODIPY-LDL –) and those that did take up LDL (BODIPY-LDL +) were selected using cell distribution on FITC-A:BODIPY Histogram and FSC-A vs FITC-A:BODIPY plots. Histogram overlay was built in FlowJo Layout editor using representative samples. For each sample median fluorescent values were calculated for BODIPY-LDL – and BODIPY-LDL + gates, median averages and standard errors across three replicates plotted with p-values calculated using One-way Anova. Additionally, population percentages were recorded in each gate for each sample, and replicate averages and SEs were plotted, p-values were calculated using One-way Anova test.

### Proteomics and drug treatment

Parental and ISG75Crc4 cells were subjected to respective 2 x EC_50_ suramin for 48 hours. Untreated and cells exposed to suramin were collected by centrifugation, washed twice with PBS containing Complete Mini Protease Inhibitor Mixture (Roche Applied Science), resuspended in 3 x NuPage LDS buffer, 2.5 x Sample Reducing Agent (Thermo Fisher Scientific) and stored at −80 °C. Treated and untreated samples were generated in triplicate. Prior to electrophoresis, samples were thawed, sonicated and aliquots containing 5 x 10^6^ cells desalted in NuPAGE Bis-Tris 4–12% gradient polyacrylamide gel (Thermo Fisher Scientific) in MOPS-SDS buffer. Following IntstantBlue (Expedeon) staining, each sample was excised. To assess cell-wide changes to the parasite proteome parental and ISG75Crc4 were processed as above, with five replicates for each cell-line. Aliquots containing 5 x 10^6^ cells were resolved on NuPAGE gels as before (Thermo Fisher Scientific) except samples were allowed to migrate further. Following IntstantBlue (Expedeon) staining each sample line was divided into three slices. All samples were subjected to tryptic digest and reductive alkylation followed by LC-MS/MS at the Fingerprints Facility (University of Dundee).

Mass spectra were analysed using MaxQuant version 1.5 [77] searching the *T. brucei brucei* 927 annotated protein database, release 39.0, supplemented with ISG75, ISG65, ISG64/65L, ISGLA and ISGLB sequences from the Lister strain 427 2018 assembly version and full GO-terms for *T. brucei brucei* 927 release 54.0 on TriTrypDB [72]. When a peptide sequence set of one protein contained the peptide set of another the two proteins were assigned to the same protein group. Output data were processed in Perseus [78]. For general ISG75Crc4 proteome analysis and only protein groups present across all relevant replicas were included. For suramin proteomics analysis, proteins and protein groups identified in 2 or more replica for each condition were included. Expression ratios between relevant sample groups were calculated as ratios of average total LFQ intensities, p-values calculated via two-sample Student's T-test on log-converted LFQ values with permutation-based FDR correction.

Fast gene set enrichment analysis (FGSEA) of the cell-wide proteome changes was performed in R using full GO-terms (Tb927 release 54.0). Protein groups were ranked via p-values (-log_10_p-value x direction of change, +1 for increased and -1 for decreased groups), and enrichment analysis was performed using FGSEA package [79] from Bioconductor 3.13 [80]. Results were plotted in R and groups indicated as enriched were exported and further manually checked for upregulation levels and significance. Proteomics data have been deposited at the ProteomeXchange Consortium via the PRIDE partner repository [81] with the data set identifier PXD031887.

### Assessment of *in vivo* proliferation

Groups of five Balb/c mice (female) were infected with parental cell-line *T. brucei* Lister 427 2T1T7Cas9 or ISG75Crc4. Log-phase trypanosomes were injected in 0.2mL in concentrations of 5 x 10^4^ and 5 x 10^2^ (10,000 and 100 cells) intraperitoneally (IP). Parasitaemia was monitored in each animal daily following infection on day three via subjective microscopic examination with a 40-X objective. Once trypanosome numbers were above five per high powered field, blood samples were collected in 1:50 and 1:100 dilutions in Hank's balanced salt solution supplemented with glucose (HBSS+G) for further quantification to determine parasite burden. Any animals showing signs of terminal illness such as non-responsive behaviour, respiratory distress or tremors were humanely killed without delay. Prior to quantifying parasitaemia blood samples were centrifuged at 800*g*, 4°C for 10 minutes, the supernatant was discarded, and pelleted blood and trypanosome cells resuspended in equal volumes of Red Blood Cell Lysis buffer (RBC, 155 mM NH_4_Cl, 10 mM NaHCO_3_, 0.1 mM EDTA), incubated for 10 minutes to allow lysis of red cells and trypanosomes counted using Neubauer haemocytometer chamber. For Western blotting, parental 2T1 and ISG75Crc4 trypanosomes were collected from terminal bleeds by centrifugation at 800*g*, 4°C for 10 minutes, resuspension in Separation Buffer (44 mM NaCl, 57 mM Na_2_HPO_4_, 3 mM KH_2_PO_4_, 55 mM glucose, pH 8.0) and passing over a DE-52 cellulose column, equilibrated in the same buffer. Trypanosomes were collected by washing the cellulose with an excess of Separation buffer, pelleted by centrifugation at 1,000*g*, 4°C for 10 minutes, quantified and lysed by addition of appropriate volumes of 4 x LDS buffer and 10 x RA buffer (Thermo Fisher Scientific). Samples were prepared and probed by Western blotting as above.

### Ethical statement on animal use

All regulated procedures on living animals were carried out under the authority of a project license issued by the Home Office under the Animals (Scientific Procedures) Act 1986, as amended in 2012 (and in compliance with EU Directive EU/2010/63). Infected animals had access to food and water *ad libitum* and were housed under a 12-hour light/dark photoperiod. Animals used in these experiments were female Balb/c mice (Harlan, UK). Any animals showing signs of terminal illness such as non-responsive behaviour, respiratory distress or tremors were humanely killed without delay.

### Data availability

Proteomics data have been deposited at the ProteomeXchange Consortium via the PRIDE partner repository [81] with the data set identifier PXD031887.

## AUTHOR CONTRIBUTION

AM, MCF and SZ conceived the work. AM, ICM and MZ carried out trypanosome work, JB performed biophysical analysis, and EP and LF were responsible for the mouse infections. AM, MCF, MZ and SZ wrote the manuscript.

## SUPPLEMENTAL MATERIAL

Click here for supplemental data file.

Click here for supplemental data file.

Click here for supplemental data file.

All supplemental data for this article are available online at www.microbialcell.com.

## Abbreviations:

DUB: – deubiquitylating enzyme,
ISG: – invariant surface glycoprotein,
ITC: – isothermal calorimetry,
LDL: – low density lipoprotein,
MFST: – major facilitator superfamily transporter,
SPR: – surface plasmon resonance,
Tet: – tetracyclin,
VSG: – variant surface glycoprotein,
WGS: – whole genome sequencing.
