# Supplementary material for: The role of invariant surface glycoprotein 75 in xenobiotic acquisition by African trypanosomes
Source: Microb Cell. 2023 Jan 27;10(2):18–35. doi: 10.15698/mic2023.02.790 (PMC9896412; doi:10.15698/mic2023.02.790)
Supplement: Supplementary file 1 [file mic-10-018-s01.pdf]

Supplementary data for:

The role of invariant surface glycoprotein 75 in xenobiotic acquisition by African trypanosomes

Alexandr Makarov[1], Jakub Began[2,†], Ileana Corvo Mautone[1,3], Erika Pinto[1], Liam Ferguson[1], Martin Zoltner[1,4], Sebastian Zoll[2,*] and Mark C. Field[1,5,*]

[1] School of Life Sciences, University of Dundee, Dundee, DD1 5EH, UK.
[2] Laboratory of Structural Parasitology, Institute of Organic Chemistry and Biochemistry, Czech Academy of Sciences, 16610 Prague 6, Czech Republic.
[3] Laboratorio de Moléculas Bioactivas, Departamento de Ciencias Biológicas, Universidad de la República, Paysandú 60000, Uruguay.
[4] Charles University, Faculty of Science, Department of Parasitology, Vestec, Czech Republic.
[5] Institute of Parasitology, Biology Centre, Czech Academy of Sciences, 37005 Ceske Budejovice, Czech Republic.
[†] Present address: Department of Biochemistry, University of Lausanne, Chemin des Boveresses 155, 1066 Epalinges, Switzerland.
* Corresponding Authors:
Mark C. Field, phone: +44(0)751 550 7880; E-mail: mfield@mac.com
Sebastian Zoll, phone: +420 220 183 591; E-mail: sebastian.zoll@uochb.cas.cz

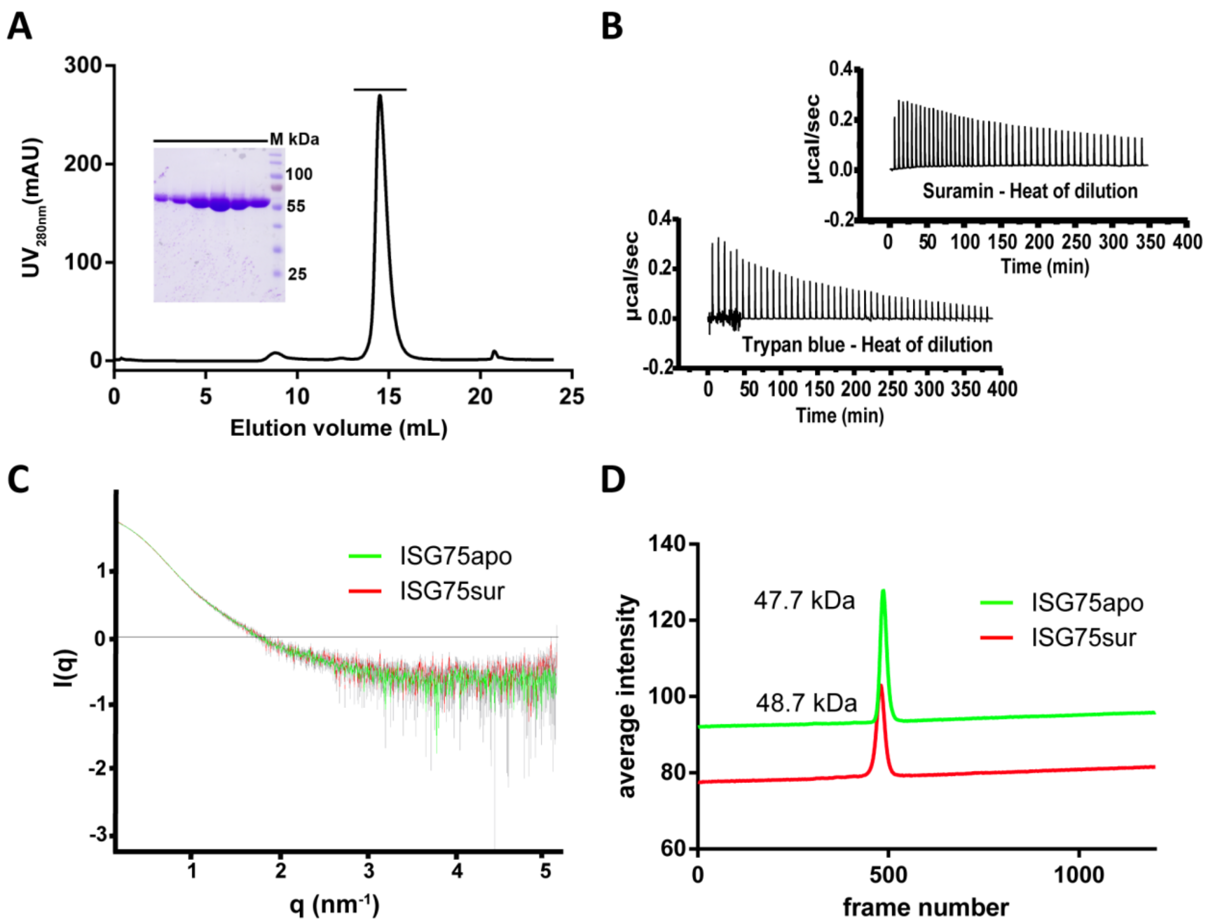

FIGURE S1. (A) Size exclusion chromatography of ISG75. Recombinant ISG75 produced in S2 insect cells elutes from a Superdex 10/300 gel filtration column as a single peak. The obtained protein is monodisperse as judged by SDS-PAGE analysis (inset). Lane M, molecular weight marker. **(B) Heat of dilution titrations for suramin and trypan blue.** 6µl aliquots of ligand at 400µM (suramin) and 500µM (trypan blue) were injected into 2.1mL of buffer. Integrated heats were subtracted from raw titrations of ligands into protein solution. **(C) ISG75apo and ISG75sur produce similar 1D SAXS curves.** Superimposed scattering curves of unliganded ISG75 (ISG75apo) and suramin-bound ISG75 (ISG75sur) are near identical, indicative of highly similar molecular shapes; I, Intensity; q, scattering vector. The experimental error is depicted in grey. **(D) ISG75apo and ISG75sur are monomers in solution.** SEC-SAXS traces of ISG75apo and ISG75sur eluting from a gel-filtration column. The molecular weight estimation for each peak is indicated. The particle mass was calculated using the Bayesian interference method. In both cases the experimentally determined values are close to the theoretical molecular weight of 49.6 kDa.

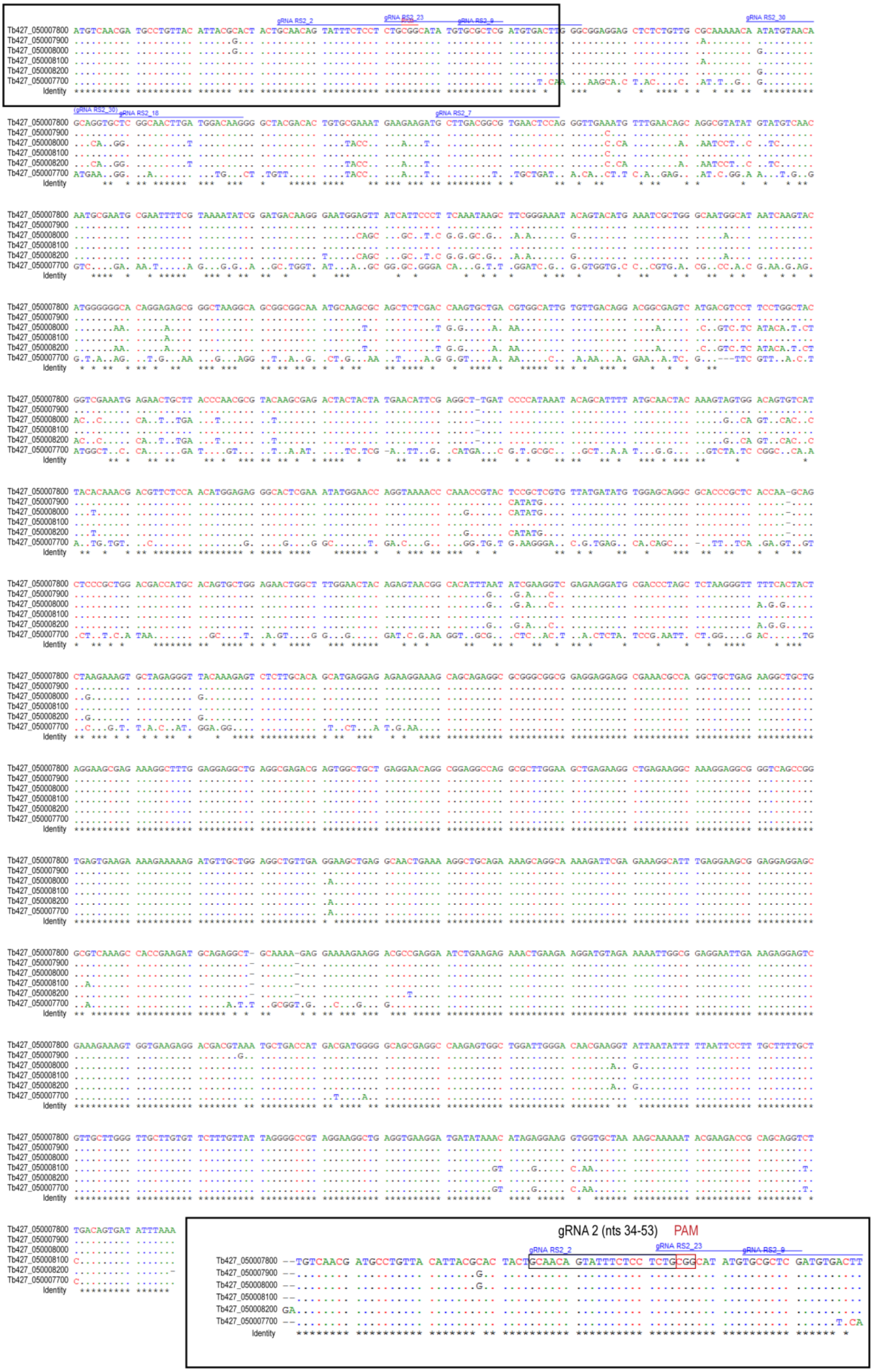

FIGURE S2. ISG75 gene alignment (top) and chosen gRNA to affect CRISR-Cas9 knockout (bottom extract window). Black and red boxes highlight respectively the gRNA sequence and its anchor CGG PAM-sequence (bottom extract window).

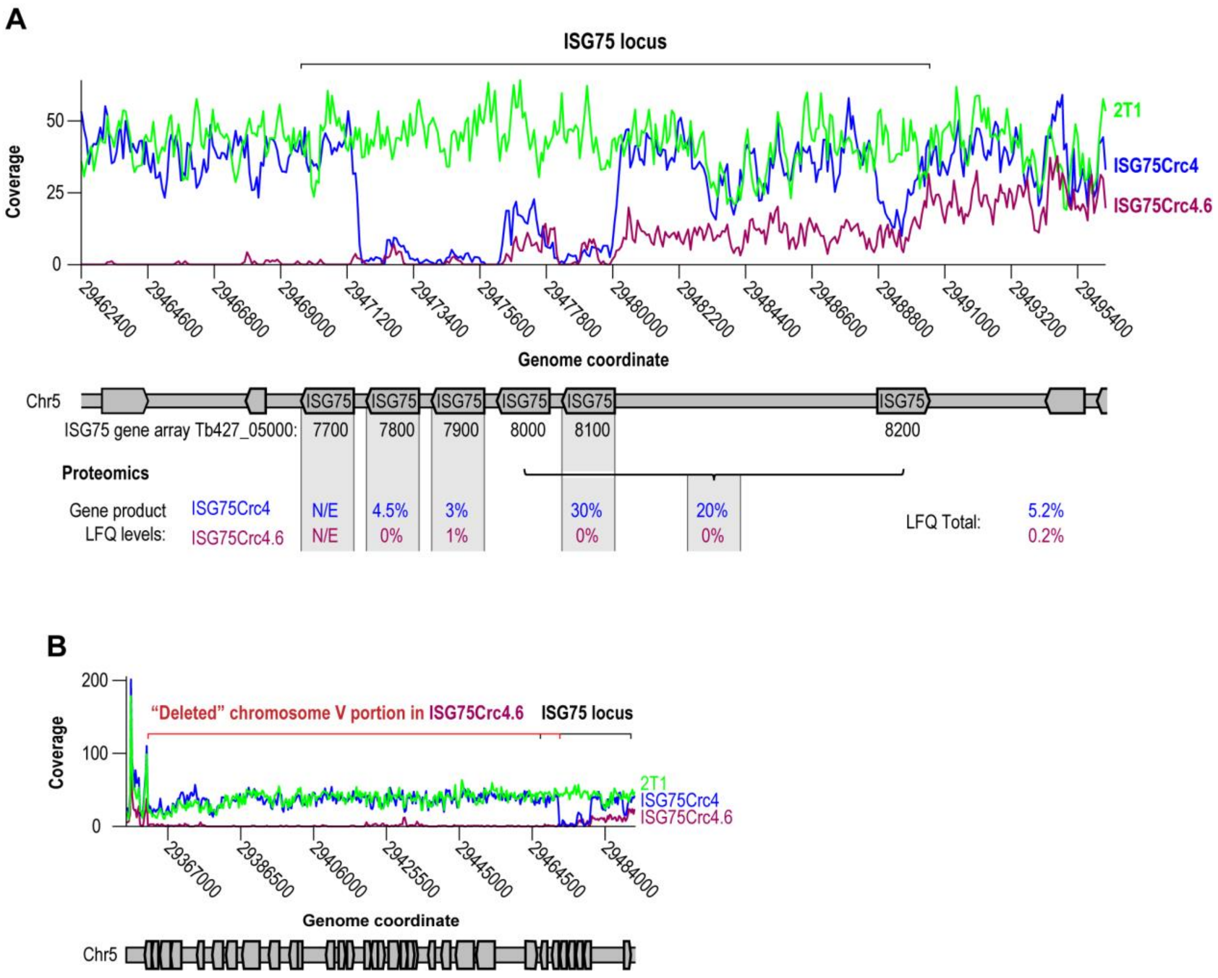

FIGURE S3. (A) Whole genome sequencing of parent, ISG75Crc4 and ISG75Crc4.6 clones. Shown are genomic reads aligned to the parental ISG75 locus (Parent 2T1 – green, ISG75Crc4 – blue, ISG75Crc4.6 – purple). Given are also whole cell proteomics LFQ values for each gene-product. Tb427_050008000 and Tb427_050008200 gene products were identified in a single protein group due to the virtual identity in protein sequence. **(B)** Whole genome sequencing reveals a large portion of the chromosome V preceding the ISG75 locus in ISG75Crc4.6n has also been edited (red bracket as indicated).

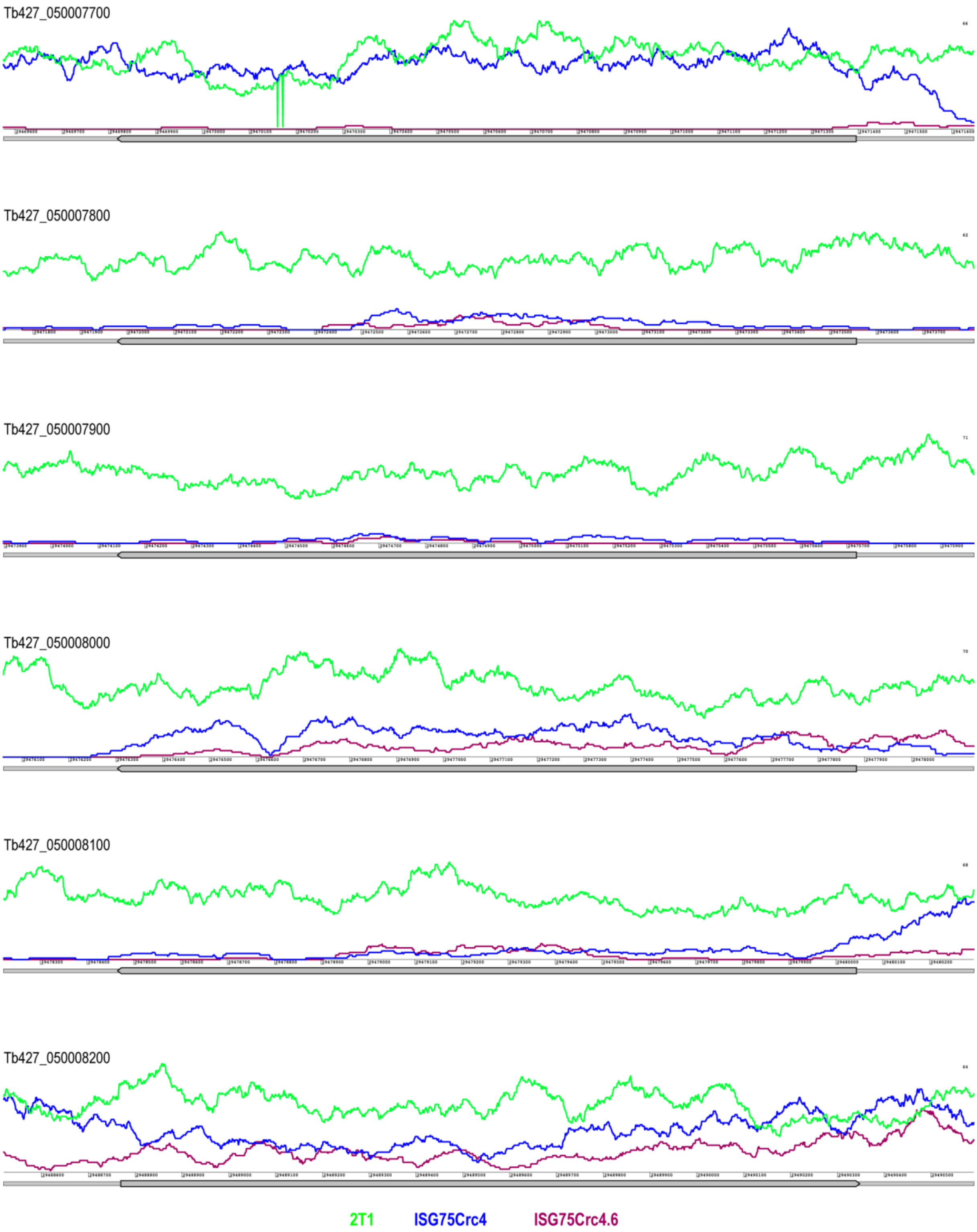

Figure S4. Detailed ISG75 gene coverage of parent, ISG75Crc4 and ISG75Crc4.6 clones in whole genome sequencing data. Shown are genomic reads aligned to the each of the ISG75 genes in the parental locus locus (Parent – green, ISG75Crc4 – blue, ISG75Crc4.6 – purple).

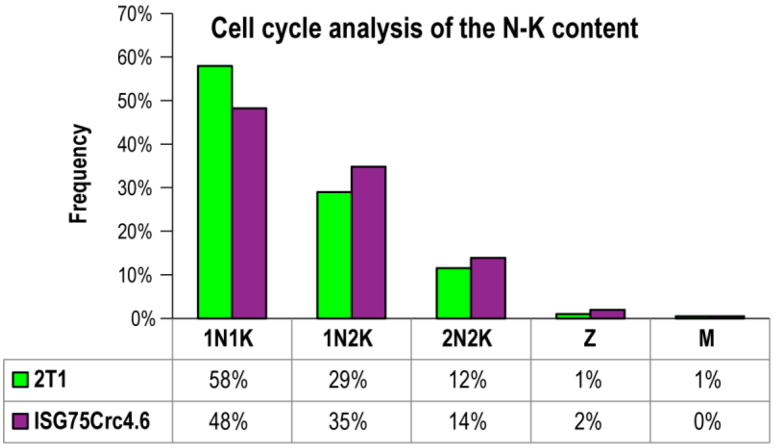

	1N1K	1N2K	2N2K	Z	M
2T1	58%	29%	12%	1%	1%
ISG75Crc4.6	48%	35%	14%	2%	0%

FIGURE S5. Bar plot showing the distribution of 2T1 (green) and ISG75Crc4 (blue) across different stages of trypanosome lifecycle, n=200. M, cells undergoing immediate mitosis. Z, monster polynucleated or cells with no nuclei.

Supplemental Tables are provided as extra xls files.

Table S1. Proteins and protein groups detected in whole cell deep proteomics of ISG75Crc4 as described in the text. Tab "ISGs in ISG75Crc4 and Crc4.6" details protein level analysis of ISG protein families in ISG75Crc4 and ISG75Crc4.6. Tab "Genes missing in ISG75Crc4.6" details proteins whose genes were excised in the process of "over-editing". Tab "Main protein groups in ISG75Crc4" details protein level analysis for ISG75Crc4. Given are protein Gene IDs, protein descriptions and protein level ratios relative to 2T1 calculated from LFQ intensities for each sample, and respective p-values calculated using via two-sample Student's T- test on log-converted LFQ values with permutation-based FDR correction. In "Main protein groups" tab rows with IDs and Descriptions highlighted in dark orange contain protein expressed 2- or more -fold of the 2T1, in light orange – proteins expressed at 1.5-2.0-fold levels of the 2T1, in red – all proteins expressed at levels below half of 2T1– all with p-values below 0.05. All insignificantly changing proteins are given in grey text. For proteins identified in FGSEA analysis respective GO Term IDs and names are given. Individual GO Term groups can be viewed by sorting in respective columns.

Table S2. Proteins and protein groups detected in suramin experiment as described in the text. Tab "Suramin Proteomics" contains protein level analysis for all protein groups identified in at least two replicas in each condition. Given are protein Gene IDs, protein descriptions and protein level ratios relative to untreated sample calculated from LFQ intensities for each sample, and respective p-values calculated using via two-sample Student's T- test on log-converted LFQ values with permutation-based FDR correction. Rows highlighted in purple contain protein previously shown as effected by suramin [37] – these can also be found in "Known Sur interactors" tab. Given also are GO Terms for relevant protein groups – glycosomal, mitochondrion and Krebs' cycle.

Supplementary methods

Small angle X-ray scattering: SAXS data were collected at ESRF, Grenoble (France), SAXS beamline BM29, using a Pilatus 2M detector (DECTRIS) and a wavelength of 0.99 Å at 20°C. For SEC-SAXS, 50 µL ISG75 at 9.8 mg/mL were injected onto a Superdex 200 3.2/300 equilibrated in 20 mM Hepes,150 mM NaCl, 3% (v/v) glycerol, pH 7.5 at a flow rate of 75 µL/min. Scattering data were acquired as components eluted from the column and passed through the SAXS measuring cell. The ATSAS software package was used to normalize the data to the intensity of the incident beam, average frames and subtract scattering contribution from the buffer. In detail, 10 frames corresponding to the void volume of the column were averaged and subtracted from ten averaged frames of the main elution peak. The particle mass was calculated using the Bayesian inference method [82].
